# Melatonin alleviates brain and peripheral tissue edema in a neonatal rat model of hypoxic-ischemic brain damage: the involvement of edema related proteins

**DOI:** 10.1186/s12887-017-0824-x

**Published:** 2017-03-28

**Authors:** Li-Xiao Xu, Yuan Lv, Yan-Hong Li, Xin Ding, Ying Wang, Xing Han, Ming-Hua Liu, Bin Sun, Xing Feng

**Affiliations:** 1 Institute of Pediatric Research, Children’s Hospital of Soochow University, Suzhou, 215006 China; 2 Department of Neonatology, Northern Jiangsu People’s Hospital, Yangzhou, 225001 China; 3 Department of Neonatology, Children’s Hospital of Soochow University, Suzhou, 215006 China

**Keywords:** Melatonin, Hypoxic-ischemic encephalopathy, Edema, Aquaporin-4, Zonula occludens-1, Occludin

## Abstract

**Background:**

Previous studies have indicated edema may be involved in the pathophysiology following hypoxic-ischemic encephalopathy (HIE), and melatonin may exhibit neuro-protection against brain insults. However, little is known regarding the mechanisms that involve the protective effects of melatonin in the brain and peripheral tissues after HIE. The present study aimed to examine the effects of melatonin on multiple organs, and the expression of edema related proteins in a neonatal rat model of hypoxic-ischemic brain damage (HIBD).

**Methods:**

One hundred ninety-two neonatal rats were randomly divided into three subgroups that underwent a sham surgery or HIBD. After the HIBD or sham-injury, the rats received an intraperitoneal injection of melatonin or an equal volume vehicle, respectively. We investigated the effects of melatonin on brain, kidney, and colon edema via histological examination and the expression of edema related proteins, including AQP-4, ZO-1 and occludin, via qPCR and western blot.

**Results:**

Our data indicated (1) Melatonin reduced the histological injury in the brain and peripheral organs induced by HIBD as assessed via H-E staining and transmission electron microscopy. (2) Melatonin alleviated the HIBD-induced cerebral edema characterized by increased brain water content. (3) HIBD induced significant changes of edema related proteins, such as AQP-4, ZO-1 and occludin, and these changes were partially reversed by melatonin treatment.

**Conclusions:**

These findings provide substantial evidence that melatonin treatment has protective effects on the brain and peripheral organs after HIBD, and the edema related proteins, AQP4, ZO-1, and occludin, may indirectly contribute tothe mechanism of the edema protection by melatonin.

## Background

Hypoxic-ischemic encephalopathy (HIE) is a leading cause of mortality and morbidity. The most frequent etiologies of it are intrapartum or late antepartum brain hypoxia and ischemia [1, 2]. 40–60% of the HIE infants were die by 2 years of age or left severe disabilities—The majority pathological mechanism of HIE is declined cerebral blood flow which results in less oxygen delivery to the brain disabilities [3] Despite the substantial research that has been conducted regarding HIE, the therapeutic interventions remain limited. Current HIE research is focused on the identification of the underlying pathophysiology of perinatal HIE and the alleviation of these pathophysiological mechanisms, especially the reversible factors [4].

Neonatal asphyxia, or birth asphyxia is caused by deprivation of oxygen to a newborn baby that usually induce edema harm to the body, frequently to the brain to result in HIE [5]. Except brain injuries, severe neonatal asphyxia is usually accompanied with multi-organ damages (such as in heart, lungs, liver, gut, kidneys) since the preferential blood supply to the brain will aggravate peripheral tissue ischemia [6, 7]. Therefore, the management of brain and peripheral tissue edema is critical for the prognosis of HIE patients. To date, a number of theories have been proposed to explain the development of edema in the brain and peripheral tissues [5, 8] Water channel aquaporins-4 (AQP-4) is specific membrane protein that have been widely investigated, which controls the influx and efflux of transmembrane water [9]. As the constituent of the continuous intercellular barrier between epithelial cells, tight junctions (TJ) are required to separate tissue spaces and regulate the selective movement of solutes across the epithelium [10]. Because zonula occludens-1 (ZO-1) and occludin are identified within TJs, together they form a virtually impermeable barrier to fluid between closely associated cells, which is very important in the maintenance of the mucosal and vascular endothelial barrier structure [11]. However, the dynamic changes in edema related proteins, such as AQP-4, ZO-1 and occludin, in the brain and peripheral tissues following HIE and their roles in edema development in central and peripheral tissues have rarely been reported. Early inhibition of edema related proteins in the brain and peripheral tissues may represent a new treatment protocol for edema following HIE.

In recent years, melatonin has been considered a promising neuro-protective drug for various acute and chronic brain injuries [12]. Melatonin is a naturally occurring hormone that is produced by the brain and was first identified to facilitate the regulation of the sleep-wake cycle [13]. Following ischemic brain injury/stroke, melatonin treatment has demonstrated a remarkable ability to reduce the infarct volume and/or inhibit neuronal cell death in different mammalian species and experimental models [12]. Moreover, it has been reported that melatonin decreased sensorimotor asymmetry and learning deficits, which thus protected pups from the long-term consequences of neonatal asphyxia [14]. In addition, histological analysis has also demonstrated melatonin treatment can increase the number of morphologically well preserved neurons in the CA1, CA2, and CA3 areas, as well as the dentate gyrus of the hippocampus in a hypoxic-ischemic mode [12]. Melatonin can decrease the cellular damage induced by epilepsy involved activation of kainate-sensitive glutamate receptors [15]. However, to the best of our knowledge, little is known regarding the mechanisms that involve the neuro-protective effects of melatonin and its effects on peripheral tissues in a hypoxic-ischemic encephalopathy model.

In the present study, we examined the effects of melatonin on brain, kidney and colon edema, and its roles in the expression of edema related proteins, including AQP-4, ZO-1 and occludin, in a neonatal rat model of hypoxic-ischemic brain damage (HIBD). We hypothesized that melatonin treatment would reduce the edema in both the brain and peripheral tissues that was induced by HIBD, at least in part, through the modulation of edema related proteins.

## Methods

### Animal preparation

192 Specific pathogen Free (SPF) Sprague Dawley (SD) rats at 7 days of age and a weight of 15–20 g, regardless of gender, were obtained from the SLRC Laboratory Animal Co. Ltd., China (License Key SCXK (Hu) 2012-0002). The neonatal rats were housed in groups of 10–15 rats per cage in standard plastic cages, and they were freely fed by female rats. The animal room was maintained on a 12-h dark-light cycle, and the temperature was maintained at 25±2°C. The protocols, which include all surgical procedures and animal usage, were approved by the Animal Care and Use Committee of Soochow University and conformed to the Guide for the Care and Use of Laboratory Animals by the National Institutes of Health.

### HIBD model establishment

HIBD was induced in the present study using a modified Rice-Vannucci method [16]. In brief, 7-day-old postnatal pups were anesthetized with is oflurane (3% in a mixture of medical air and oxygen, 70:30 ratio). The left common carotid artery of each pup was identified, exposed, and permanently ligated with 5-0 surgical silk via a near-midline incision. The wound was closed, and the pups were allowed to recover from the anesthesia after the procedure, which lasted approximately 5 min per pup. Following recovery with their dams for 2 h, the pups were subsequently placed in a jar perfused with a humidified and pre-warmed gas mixture (8% oxygen balanced with nitrogen) for 2.5 h. A constant temperature of 37 °C was maintained throughout all procedures. Following the hypoxia, the animals were returned to their dams, and the ambient temperature was maintained at 37 °C for 24 h. The sham animals underwent anesthesia, and the common carotid artery was exposed without ligation or hypoxia.

### Experimental protocol

The animals were randomly allocated into three subgroups (*n* = 64 per group) by throwing a 9-faces dice: (A) the numbers 1, 4, 7 were in sham group (Sham group), (B) the numbers of 2, 5, 8 were in HIBD group (Mod group), and (C) the numbers of 3, 6, 9 were in HIBD + melatonin group (MT group). Immediately after the HIBD injury, the rats received an intraperitoneal injection of melatonin (10 mg/kg body weight) (Sigma, USA) in the MT group or an equal volume of vehicle in the Sham and Mod Groups.

### Cerebral edema measurement

Cerebral edema was determined via brain water content (BWC) measurement using the wet weight—dry weight technique, and the results are expressed as a percentage of the water content [17].

### Hematoxylin& eosin (H-E) staining of the brain, kidney and colon

The rats were sacrificed under deep anesthesia at 3, 6, 24 and 72 h after HIBD or sham-injury for H-E staining. The rats were perfused with heparinized saline and 4% neutral buffered formalin. The same cortical layer of left frontal cortex, renal cortex and colon were dissected. 5 μm thick paraffin-embedded sections were prepared for H-E staining.

### Quantification of the swelling cell

For quantification analysis, the swelling cell in the H-E stained cortex, renal cortex and colon samples were defined as the cells which were abnormal enlargement of a body, transparent cytosol, light stained, and even balloon-like structures. The total number of the swelling glial cell, renal tubular epithelial cells, or colon epithelial cells were countered in 5 random fields under high magnification view. The swellingcells of cerebral glial, glomerular epithelial, colon epithelial cells were quantified by dividing to the corresponding total cells.

### Transmission electron microscopy (TEM) of the brain, kidney and colon

The rats were sacrificed under deep anesthesia at 3, 6, 24 and 72 h after HIBD or sham-injury for ultrastructure examination using TEM. Slices (approximately 1 mm thick) of the same cortical layer of left frontal cortex, renal cortex and colon were placed into cold 4% glutaraldehyde solution immediately after harvest and maintained at less than 4°C. The whole process of sampling required less than 3 min. Semi-thin sections were cut from these slices, rinsed overnight in 0.1 M phosphate buffer, post-fixed for 2 h in 1% osmium tetroxide, dehydrated and then embedded in Araldite mixture. Ultrathin sections stained in uranyl acetate and lead citrate were viewed in a Philips CM 120 electron microscope (Worcester, MA, USA).

### Real time-quantitative polymerase chain reaction (RT-qPCR) for AQP4, ZO-1, and occludin

The rats in the three groups were sacrificed under deep anesthesia at 3, 6, 24 and 72 h after HIBD or sham-injury for RT-qPCR [18]. The rats were transcardially perfused with 250 ml of cold heparinized 0.9% saline, and the left frontal cortex, renal cortex and colon were rapidly removed and stored in liquid nitrogen immediately until use. The AQP-4, ZO-1, and occluding mRNA levels were determined using RT-qPCR. The primer sequences and PCR conditions were as follows: AQP-4: forward, 5’- cggttcatggaaacctcact -3’; reverse, 5’- catgctggctccggtataat -3’; Zo-1: forward, 5’- gtatccgattgttgtgttcc -3’; reverse, 5’- tcacttgtagcaccatccgc -3’; occludin: forward, 5’- cacgttcgaccaatgc -3’; reverse, 5’- cccgttccataggctc -3’; and β-actin: forward, 5’-cccatctatgagggttacgc -3’; reverse, 5’- tttaatgtcacgcacgatttc -3’. RT-qPCR analysis was performed using the iQ5™ Real-Time PCR System (Bio-Rad, USA), which applied real-time SYBR Green PCR technology. All samples were analyzed in triplicate. β-actin was selected as an acceptable endogenous reference “housekeeping” gene. The relative change in the target cytokine mRNA expression was determined by the following equation: fold change = 2 − [ΔΔCt], where the Ct value is the cycle number at which the fluorescence signal crosses the threshold.

### Western blot

0.5–1 g of the mouse cortex, renal cortex, and colon were collected and lysed in 500 μl of homogenization buffer. Each 30 μg of the extracted protein was separated on 10% SDS-PAGE gels and transferred onto a nitrocellulose membrane. Subsequently, the membrane was reacted with mouse monoclonal AQP-4 (all antibodies are from Abcam;Cambridge,MA),mouse monoclonal ZO-1, mouse monoclonal occluding, and mouse monoclonal GAPDH. The probed membrane was then incubated with horseradish peroxidase-conjugated rabbit anti-mouse IgG. The probed proteins (AQP-4, ZO-1, occluding, and GAPDH) were visualized by enhanced chemiluminescent.

### Statistical analysis

SPSS 16.0 software was used for the statistical analyses (SPSS, Inc., Chicago, IL, USA). All data are presented as the mean ± SD. The data were subjected to a one-way analysis of variance followed by a Student-Newman-Keuls test. Statistical significance was determined as *P* < 0.05.

## Results

### Cerebral edema measurement

**Fig. 1:**
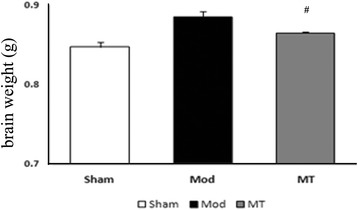
Effects of melatonin treatment on the BWC (*n* = 6 per group). HIBD could result in a significant increase in the BWC in comparison with the one in the sham rats (*P* < 0.05), which was significantly attenuated following melatonin treatment (*P* < 0.05)

Twenty-four h after HIBD, a significant increase in the BWC, which indicated cerebral edema, was identified in the cerebral cortex compared with the sham rats that received vehicle treatment (*P* < 0.05; Fig. 1). The BWC increase induced by HIBD was significantly attenuated in the HIBD rats following melatonin treatment (*P* < 0.05; Fig. 1), which indicates an improvement in cerebral edema.

### Edema estimation of the brain, kidney and colon by H-E staining and edema cell quantification

**Fig. 2:**
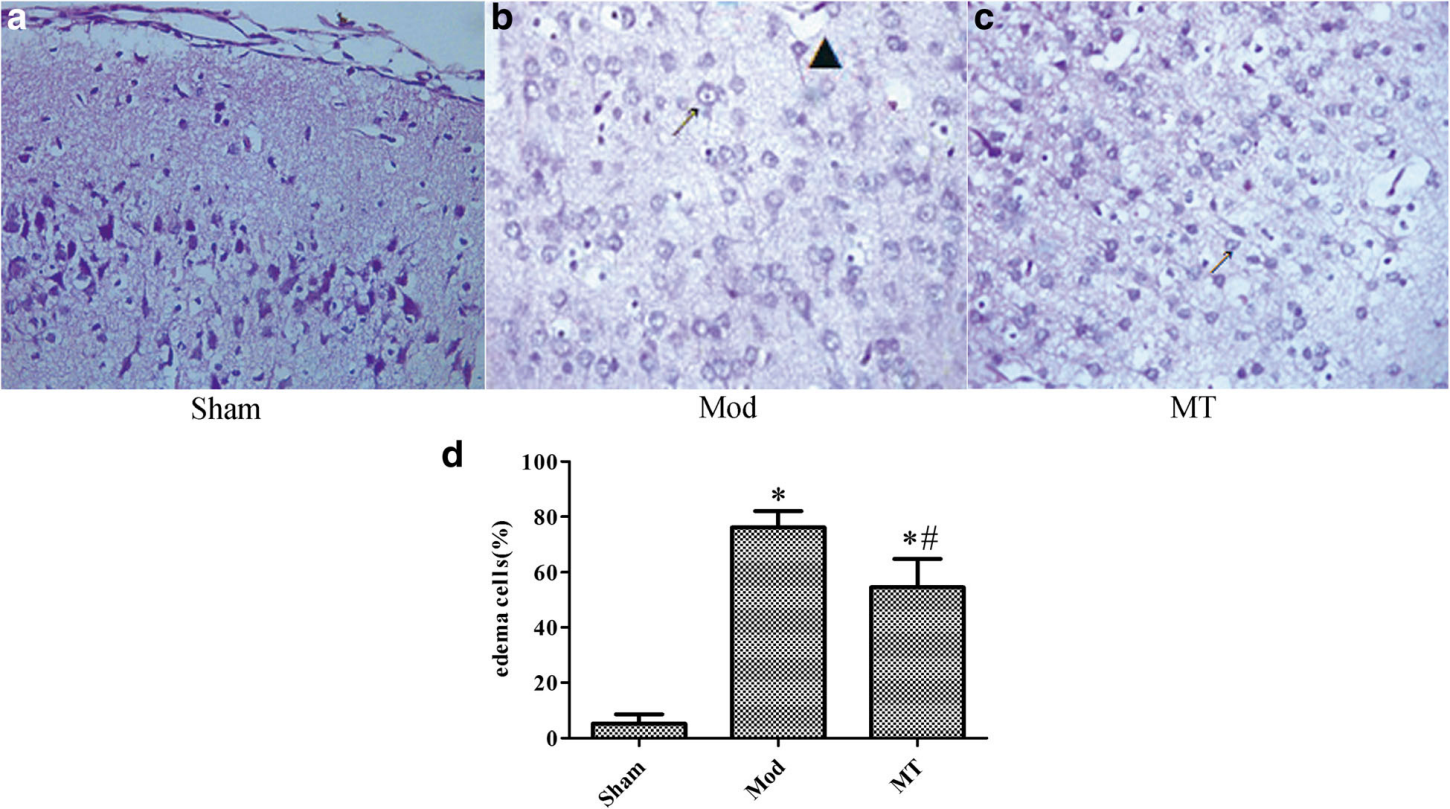
Cerebral cortex under a 400 × light microscope after H-E staining. 24 h treatment of the HIBD results in insignificant glial cell swelling and karyopyknosis in combination with interstitial tissue edema, capillary contracture and blank zones around the capillary in H-E stained samples (**a** and **b**), which was improved by melatonin treatment (**c**). Furthermore, **d** showed the quantification analysis of the swollen glial cells in the total glial cells in the indicated groups.↗ represents cytotoxic edema, and ▲ represents vasogenic edema

**Fig. 3:**
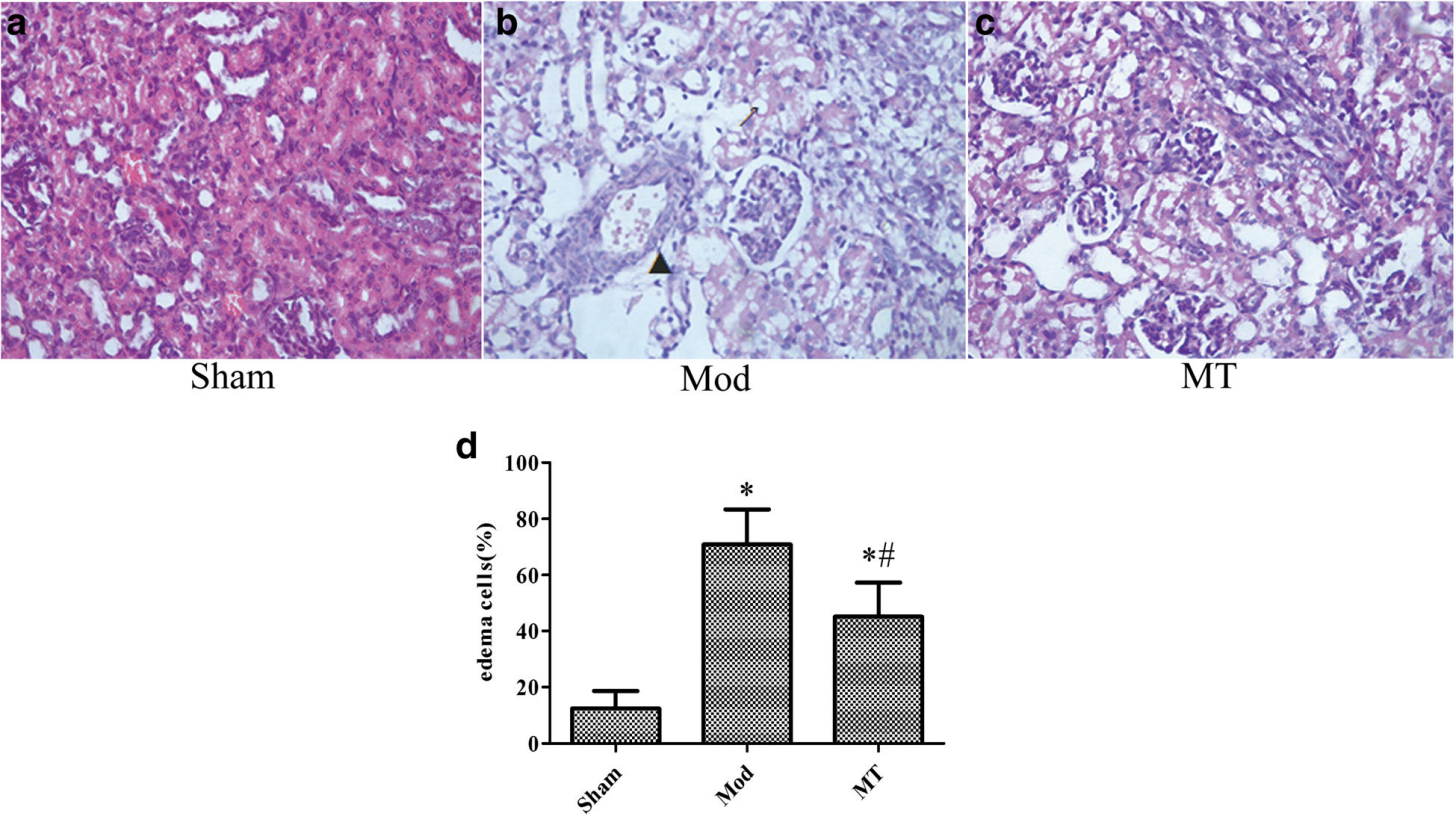
Renal cortex under a 400 × light microscope after H-E staining. Similar treatment as Fig. 2 induced significant swelling of the renal tubular epithelial cells and interstitial tissue edema, which was improved by melatonin treatment in H-Estained samples (**a** and **b**). However, there were few changes regarding the glomeruli in these groups. In addition, the swollen renal tubular epithelial cells were quantified in the total renal tubular epithelial cells in the indicated groups.↗ represents cytotoxic edema, and▲ represents vasogenic edema

**Fig. 4:**
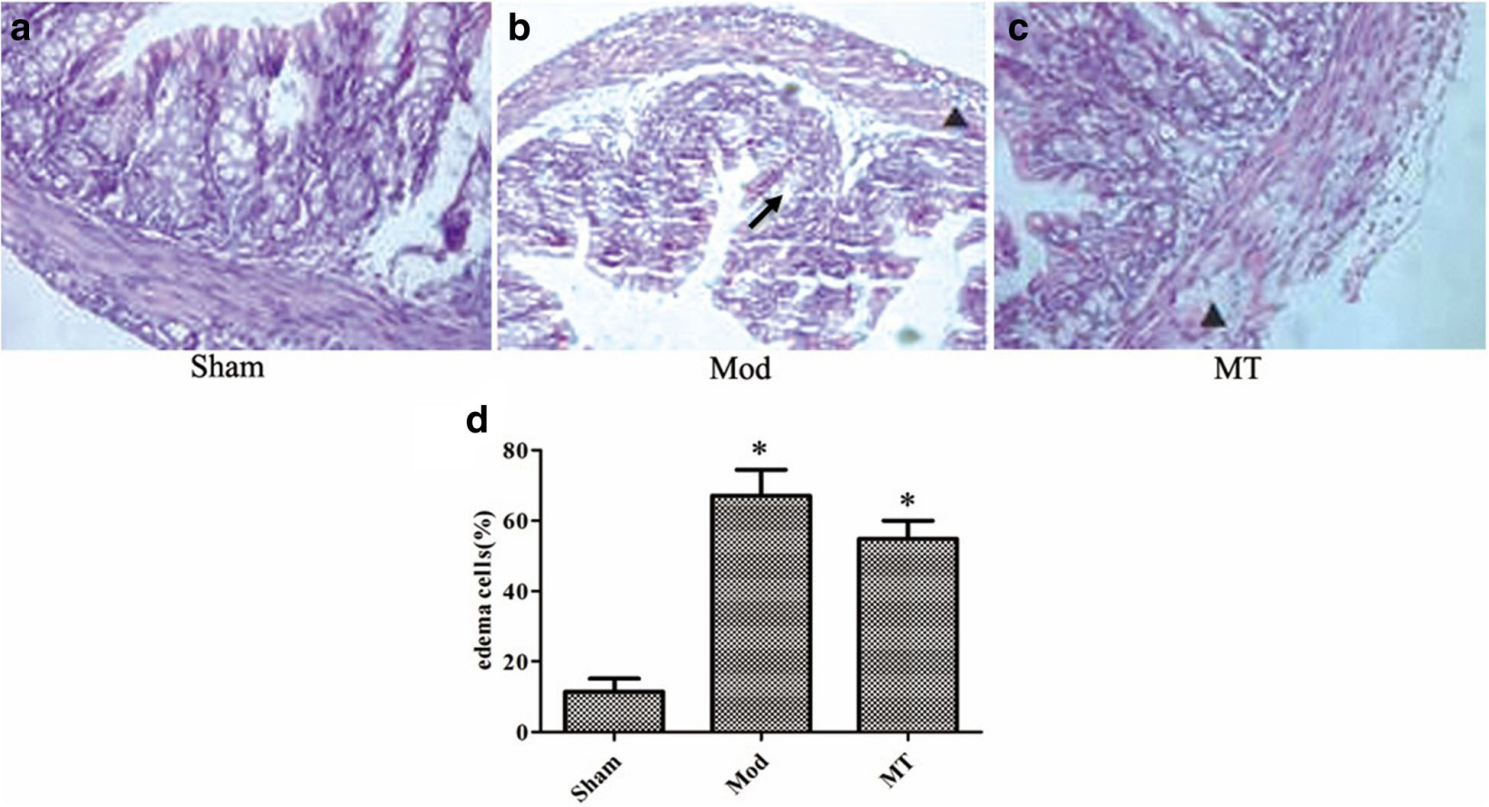
Colon under a 400 × light microscope after H-E staining. 24 h-HIBD persuaded significant edema and even ballooning degeneration of the renal tubular epithelial cells (**b**) compared with sham group (**a**), and these alterations were improved by melatonin treatment (**c**). The edema colon epithelial cells were quantified as well in the total colon epithelial cells in the indicated groups (**d**). ↗ represents cytotoxic edema, and▲ represents vasogenic edema

In the Sham group, the cellular morphologies were normal in the brain (Fig. 2), kidney (Fig. 3) and colon (Fig. 4). In the Mod group, there was significant glial cell swelling and karyopyknosis in combination with interstitial tissue edema, capillary contracture and blank zones around the capillary (Fig. 2). And a significant swelling of the renal tubular epithelial cells was identified, as well as interstitial tissue edema (Fig. 3). Moreover, significant edema and even ballooning degeneration of the renal tubular epithelial cells were identified (Fig. 4). In the MT group, there was an improvement in the edema compared with the Mod group (Figs. 2, 3 and 4). In addition, the corresponding edema cells were quantified (Fig. 2d, 3d, and 4d) which is consistent with the observation above.

### TEM of the brain, kidney and colon

**Fig. 5:**
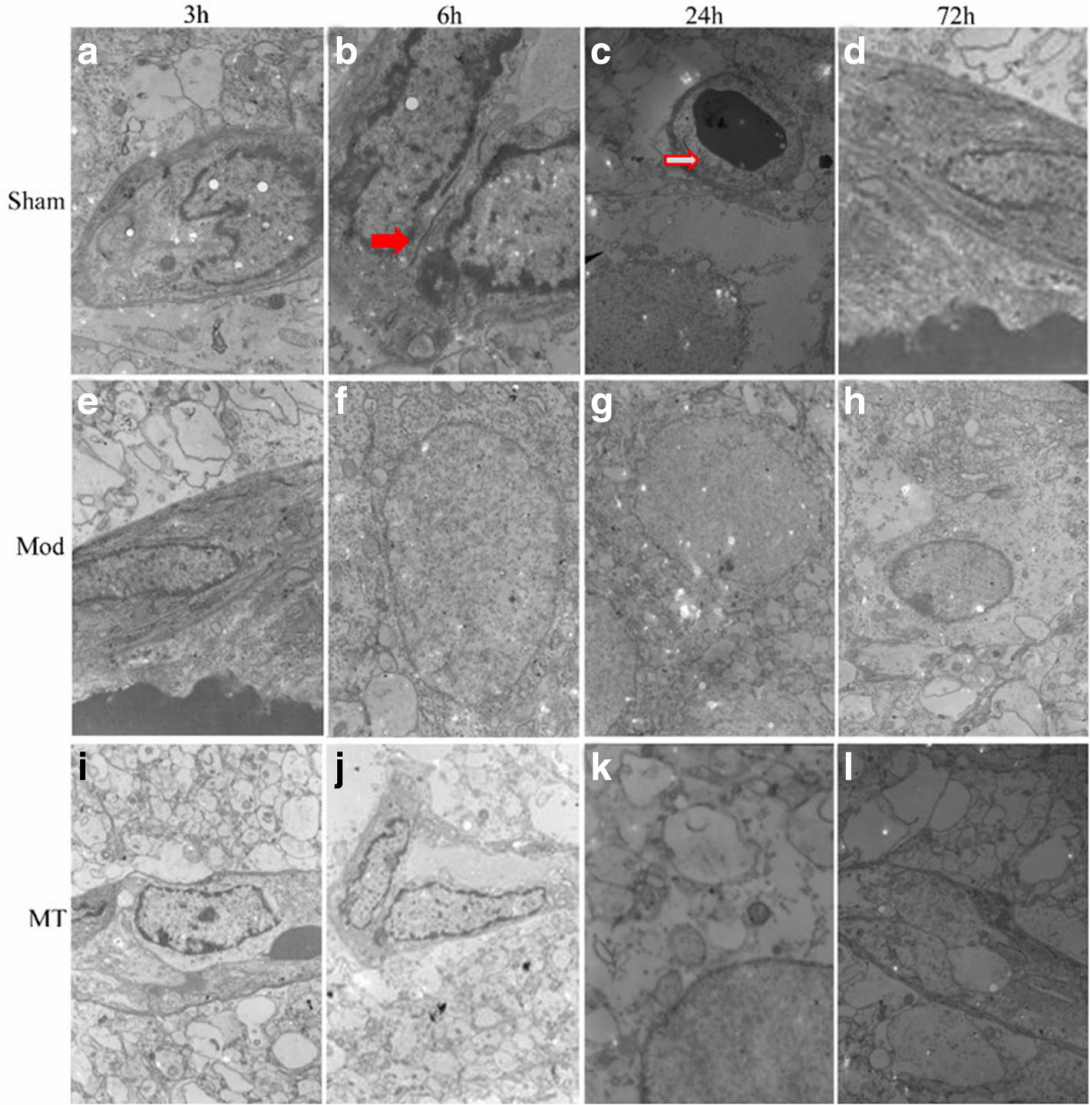
Cerebral glial cells and capillaries assessed via a transmission electron microscope. HIBD could result in a remarkable pathologic change at 24 h after injury, including clearly decreased organelles and their extraordinary swelling, fused or disappeared intercellular junctions and additional stenosis and contracture of the capillary lumens (**e-h**) compared with sham group (**a-d**). Melatonin treatment could reduce the severity of pathology after injury (**i-l**). Red solid arrows represent tight junctions, red hollow arrows represent capillary lumens, and ▲ represents cell vacuoles

**Fig. 6:**
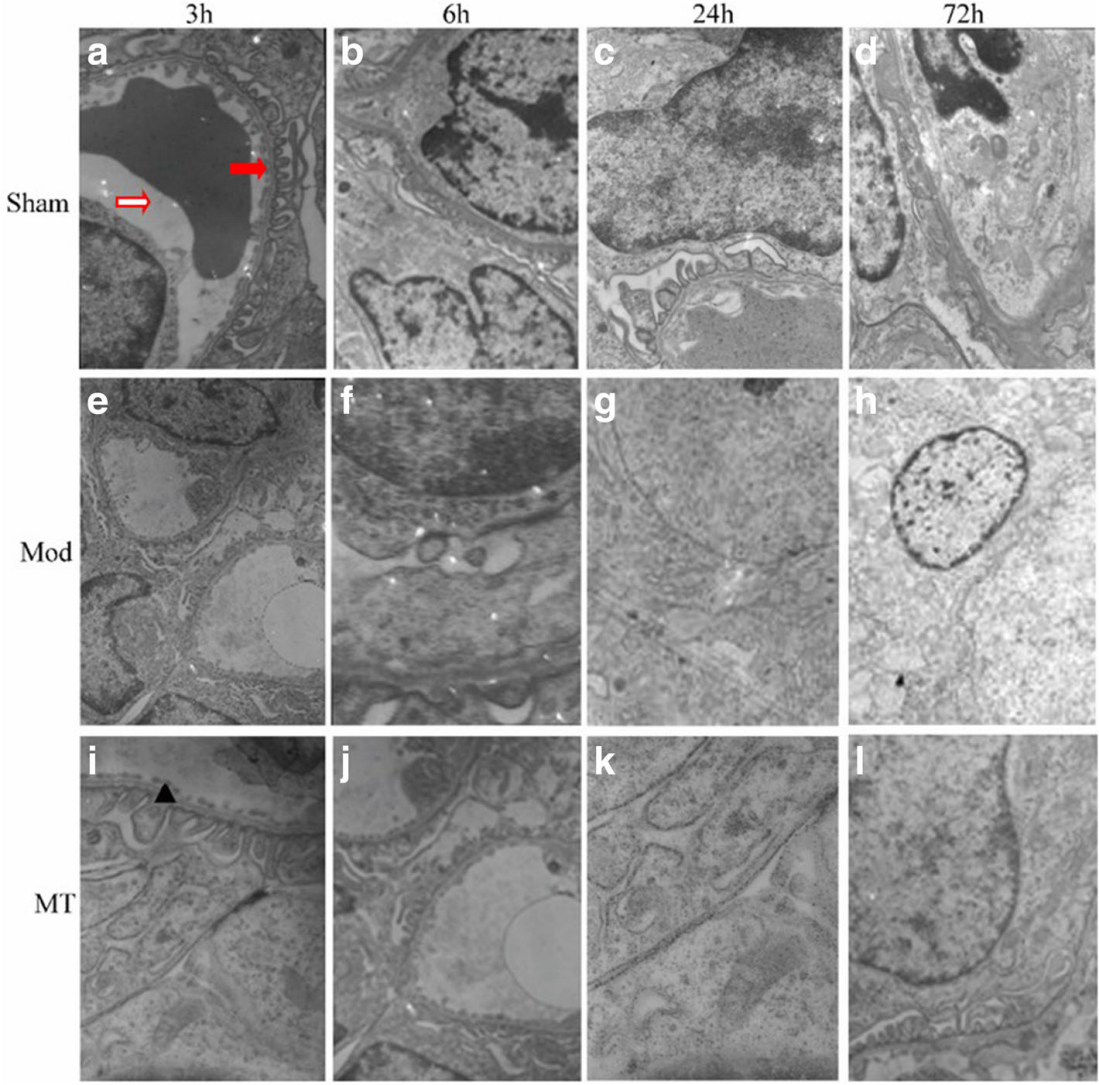
Glomerular filtration barriers assessed via a transmission electron microscope. HIBD could result in a remarkable damage in the membrane and foot processes at 24h after injury, including vague membranes and randomly arranged and further fused foot process (**e-h**) compared with sham group (**a-d**). Melatonin treatment could reduce the severity of pathology after injury (**i-l**). Red solid arrows represent the foot process, red hollow arrows represent capillary lumens, and ▲ represents filtration barrier epithelial cells

**Fig. 7:**
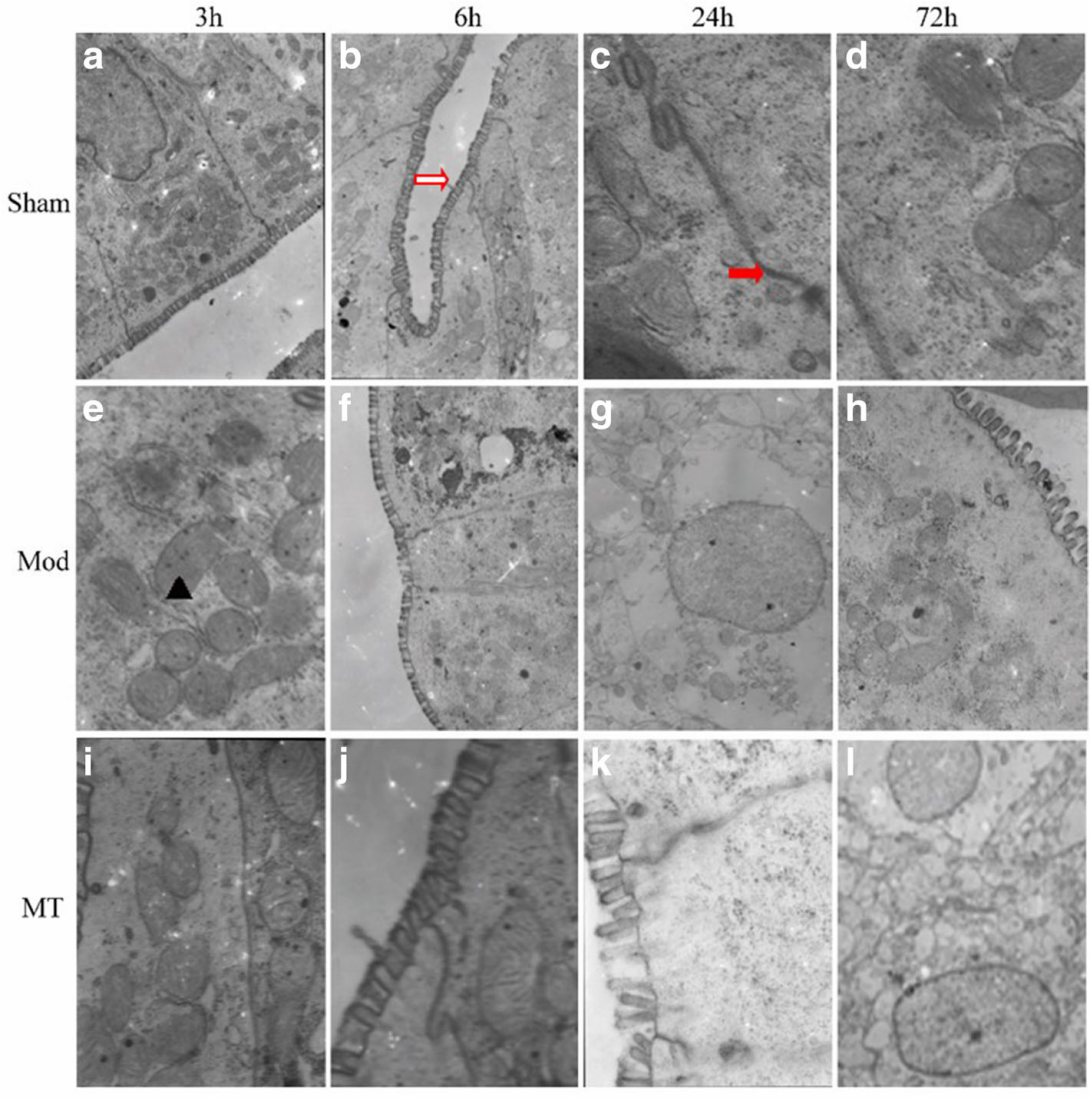
Colon epithelial cells assessed via atransmission electron microscope. HIBD could result in edematous changes in the matrix, cristae in the mitochondria, the sparse distributed swelled microvilli, the vague epithelial cell organelles, remarkable decreased junctions and the loosened interstitial tissue (**e-h**) compared with sham group (**ad**). Melatonin treatment could reduce the severity of pathology after injury (**i-l**). Red solid arrows represent tight junctions, red hollow arrows represent intestinal epithelium microvilli, and ▲ represents mitochondria

In the Sham group, the cellular morphologies were normal in the brain (Fig. 5), kidney (Fig. 6) and colon (Fig. 7). In the Mod group, at 3 h after HIBD, there was a minimally dilated rough endoplasmic reticulum (ER) and edematous changes in the matrix and cristae of the mitochondria (Fig. 5). And the tubular epithelial cells were slightly swollen (Fig. 6). Moreover, the microvilli were sparse, and the rough ER was slightly dilated (Fig. 7). At 6 h after HIBD, there were more substantial edematous changes in the organelles and vacuolation of the mitochondria, as well as fusion in some intercellular junctions (Fig. 5). And the capillary lumen narrowed while the epithelial hole increased (Fig. 6). Moreover, the edematous changes in the colon cells progressed, and the junctions between the cells became somewhat vague (Fig. 7). A remarkable pathologic change occurred at 24 h after HIBD, which was characterized by clearly decreased organelles and their extraordinary swelling. Many intercellular junctions had fused or disappeared (Fig. 5). And there was a vague membrane, which made it difficult to distinguish the three layers, and the foot process was randomly arranged and further fused (Fig. 6). In the colon (Fig. 7), even the sparse distributed microvilli swelled, and the epithelial cell organelles were vague. 72 h after the operation, the situation had improved in the brain (Fig. 5), kidney(Fig. 6) and colon(Fig. 7). In the MT group, the time course of edema was similar compared with the Mod group, but the severity was reduced (Figs. 5, 6 and 7).

### AQP-4 expression in the cerebral cortex, renal cortex and colon

**Fig. 8:**
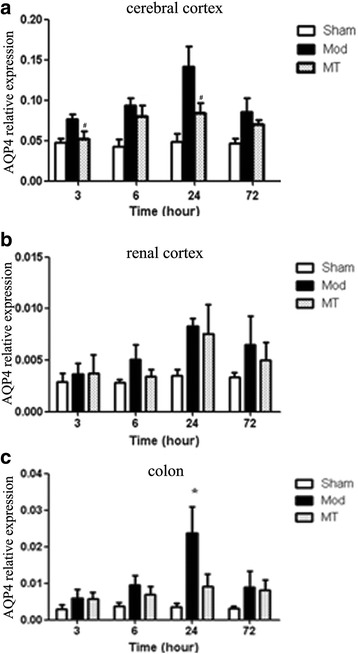
Real time-qPCR for AQP-4 in the cerebral cortex (**a**), renal cortex (**b**) and colon (**c**). HIBD induced a significant increase in the AQP-4 mRNA expression in the cerebral cortex and colon. Melatonin treatment significantly decreased the AQP-4 mRNA expression in comparison with the one without melatonin treatment in HIBD group. The data represent the mean ± SD

As shown in Fig. 8a, HIBD induced a significant increase in the AQP-4 mRNA expression in the cerebral cortex compared with the sham injury at 3, 6 and 24 h after injury (*P* < 0.05). Melatonin treatment significantly decreased the AQP-4 mRNA expression in the cerebral cortex compared with the Mod Group at 3 and 24 h after the injury (*P* < 0.05). Furthermore, the data in Fig. 8b indicated that there was no significant difference in the AQP-4 mRNA expression in the renal cortex between the three groups at any time-point (*P* > 0.05). In addition, the data in Fig. 8c indicated that HIBD induced a significant increase in the AQP-4 mRNA expression in the colon compared with the sham injury only at 24 h after the injury (*P* < 0.05). The mRNA changes in the cerebral cortex and renal cortex after 24 h HIBD treatment were verified as well by western blot (Fig. 11a and b). AQP-1 protein expression of the cerebral cortex and renal cortex were augmented significantly 24 h after HIBD and melatonin partially (cerebral cortex, Fig. 11a) or completely (renal cortex, Fig. 11b) prevented this increase. The detection of the protein expression in the colon samples did not succeed (data did not show).

### ZO-1 expression in the cerebral cortex, renal cortex and colon

**Fig. 9:**
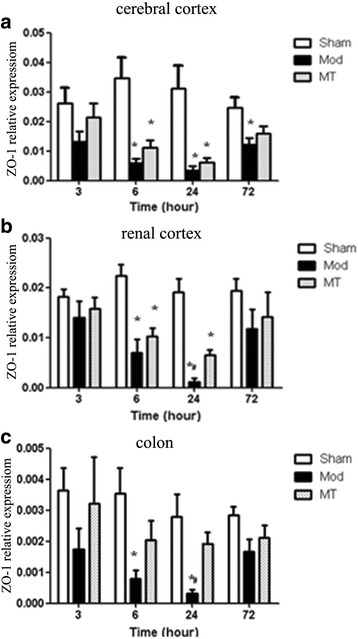
Real time-qPCR for ZO-1 in the cerebral cortex (**a**), renal cortex (**b**) and colon (**c**). HIBD induced a significant decrease in the ZO-1 mRNA expression in the cerebral cortex, renal cortex and colon. Melatonin treatment could significantly increase the ZO-1 mRNA expression in the renal cortex and colon in comparison with the one without melatonin treatment in HIBD group. The data represent the mean ± SD

As shown in Fig. 9a, HIBD induced a significant decrease in the ZO-1 mRNA expression in the cerebral cortex compared with the sham injury at 6, 24 and 72 h after the injury (*P* < 0.05). There was no significant difference in the ZO-1 mRNA expression in the cerebral cortex between the Mod and MT groups at any time-point (*P* > 0.05). Furthermore, the data in Fig. 9b indicated that the ZO-1 mRNA expression in the renal cortex was significantly decreased compared with the sham injury at 6 and 24 h after the injury (*P* < 0.05). Melatonin significantly increased the ZO-1 mRNA expression in the renal cortex compared with the Mod group at 24 h after the injury (*P* < 0.05). In addition, the data in Fig. 9c indicated that HIBD induced a significant decrease in the ZO-1 mRNA expression in the colon compared with the sham injury at 6 and 24 h after the injury (*P* < 0.05), whereas melatonin treatment significantly increased the ZO-1 mRNA expression in the colon compared with the Mod group24 h after the injury (*P* < 0.05). The findings in the cerebral cortex and renal cortex at 24 h after injury were further confirmed by western blot (Fig. 11a and b). ZO-1 protein of the cerebral cortex has declined 24 h after HIBD and melatonin turn back this response significantly (Fig. 11a). Likewise, ZO-1 protein expression of the renal cortex has slightly weakened 24 h after HIBD and this decrease was prohibited considerably by melatonin treatment (Fig. 11b).

### Occludin expression in the cerebral cortex, renal cortex and colon

**Fig. 10:**
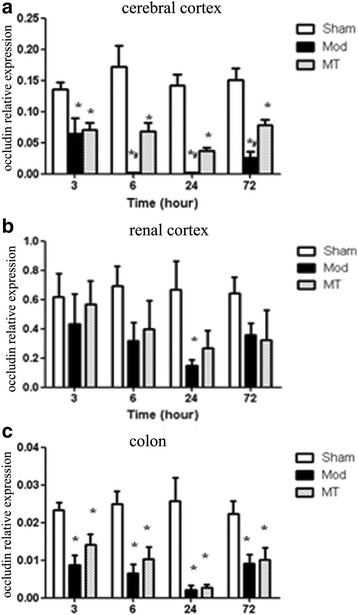
Real time-qPCR for occluding in the cerebral cortex (**a**), renal cortex (**b**) and colon (**c**). HIBD induced a significant decrease in the occluding mRNA expression in the cerebral cortex, renal cortex and colon, which could be significantly increased by melatonin treatment. The data represent the mean ± SD

**Fig. 11:**
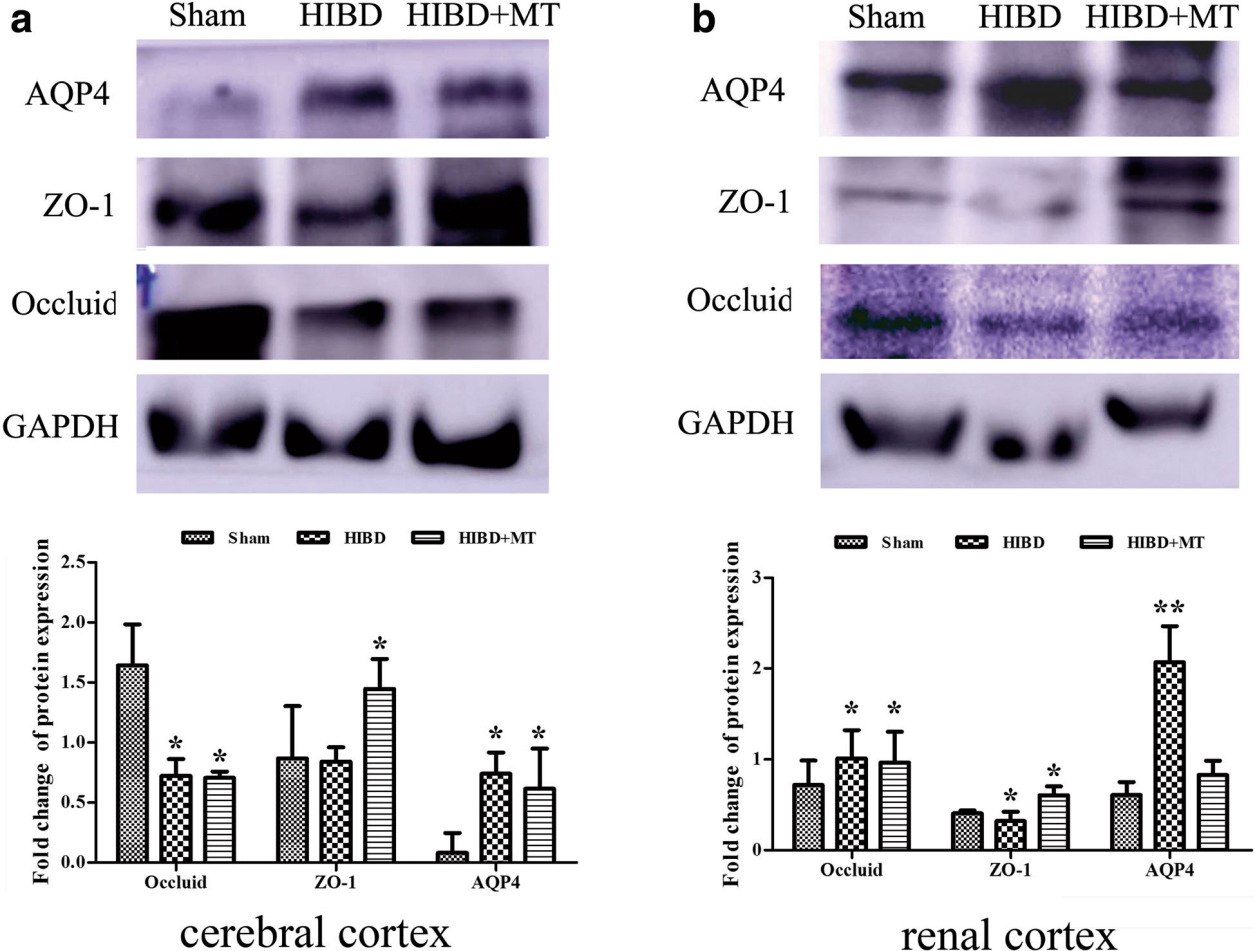
Western blot of the APQ-4, ZO-1, and occludin inthe cerebral cortex, renal cortex. The protein expression of the APQ-4, ZO-1, and occluding were detected by western blot after 24 h HIBD in cerebral cortex (**a**), renal cortex (**b**). The targeted protein was indicated in the left of the images and the treatment conditions were shown on the top of the pictures. GAPDH were used as a housekeeping gene references. The shown pictures represented three other independent results. *P < 0.05, compared with the sham operation group

As shown in Fig. 10a, HIBD induced a significant decrease in the occluding mRNA expression in the cerebral cortex compared with the sham injury at each time-point after the injury (*P* < 0.05). Melatonin treatment significantly increased the occluding mRNA expression in the cerebral cortex compared with the Mod group at 6, 24 and 72 h after the injury (*P* < 0.05). Moreover, the data in Fig. 10b indicated that the occluding mRNA expression in the renal cortex was significantly decreased compared with the sham injury 24 h after the injury (*P* < 0.05). There was no significant difference in the occluding mRNA expression in the renal cortex between the Mod and MT groups at any time-point (*P* > 0.05). In addition, the data in Fig. 10c indicated that HIBD induced a significant decrease in the occluding mRNA expression in the colon compared with the sham injury at each time-point after the injury (*P* < 0.05), whereas there was no significant difference in the occluding mRNA expression in the colon between the Mod and MT groups at any time-point (*P* > 0.05). In addition, these results in the cerebral cortex and renal cortex after 24 h HIBD were proven by western blot (Fig. 11a and b). Theoccludin protein expression of the cerebral cortex and renal cortex have decreased significantly 24 h after HIBD and melatonin partially attenuated these changes.

## Discussion

In the present study, we examined the effects of melatonin on brain, kidney and colon edema, and its roles in the expression of edema related proteins in a neonatal rat model of HIBD. The main findings are as follows: (1) Melatonin reduced the histological injuries of the brain and peripheral organs, which were induced by HIBD, as assessed via H-E staining and TEM. (2) Melatonin alleviated the HIBD-induced cerebral edema characterized by the decreased BWC. (3) HIBD induced significant changes in the mRNA expression of edema related proteins, including AQP-4, ZO-1 and occludin, which were partially reversed by melatonin treatment.

AQP-4 is one of the most deeply lucubrated edema related proteins in the central nervous system, especially regarding cytotoxic cerebral edema [19]. The expression of AQP-4 is positively related to edema at an early stage after HIBD insults in rats with a genetic knockout of AQP-4 [20], which is consistent with our findings that cerebral edema characterized by increased BWC is accompanied by significantly increased AQP-4 expression in the cerebral cortex following HIBD insults. Moreover, we demonstrated that AQP-4 mRNA expression increased as early as 3 h after HIBD injury and was accompanied by the histological signs of cerebral edema, such as mitochondrial swelling, rough ER expansion, and capillary contracture surrounded by an electronic blank area, when examined by TEM, these alterations represented both cytotoxic edema and vasogenic edema. The present study demonstrated that AQP-4 mRNA expression reached its peak at 24 h after injury and subsequently decreased, which was accompanied by similar changes assessed via TEM.

Acute kidney injury typically presents with edema and necrosis, and the renal tubules exhibit an earlier and more severe manifestation [21]. In the present study, we identified obvious edematous and necrotic changes together with interstitial edema. However, AQP-4 mRNA expression was not significantly different after hypoxic and ischemic damage, which indicates that there may be no obvious correlation between AQP4 and renal tubular epithelial cell and interstitial tissue edema. Nevertheless, based on evidence regarding the organic distribution of AQPs and the existence of more than one subtype of AQPs positioned in the kidney [22] we speculated that tubular cell edemamay be affected by other AQP subtypes; thus

Additional research is required to verify this hypothesis. Neonates with HIE typically exhibit abnormal manifestations in the digestive tract, such as gastrointestinal bleeding and feeding intolerance, and the most severe symptom is necrotizing enterocolitis. The ileum and colon are more likely to be affected because of their special blood supply characteristics and molecular basis. Previous studies have demonstrated that AQP-4 is distributed in the entire colon, mainly on the top of the villi epithelium and lumen [23]. However, little is known regarding the relationship between AQP-4 and hypoxic-ischemic intestinal damage. In our experiment, we identified colon epithelial cell edema using TEM, as well as an increased AQP-4 expression, which is consistent with the extent of edema in the colon. Thus, we could infer that AQP-4 expression may play a role in hypoxic-ischemic colon damage.

Tight junction proteins (TJPs) represent a series of proteins closely related to the blood-brain barrier (BBB) in the brain. In our experiment, we investigated two types of TJPs, including ZO-1 and occludin. The expression of ZO-1 and occluding mRNA were decreased as early as 3 h after HIBD injury, which was accompanied by signs of vasogenic edema in the brain assessed with TEM. This finding further verifies that TJPs are negatively related to edema. Tight junctions are also very important for the maintenance of epithelial homeostasis. Previous studies have indicated that TJPs are expressed on tubules [24]. Thus, the significant decrease in TJP expression, especially ZO-1 expression, may be related to the damage of tight junctions between tubules and renal interstitial, which tends to lead to vasogenic edema. In addition, the use of TEM in the present study demonstrated the existence of colon epithelial edema as early as 3 h after HIBD, and the edema peaked 24 h after injury, which was accompanied by significantly decreased ZO-1 and occluding mRNA expression in the colon. Thus, the damaged tight junction structure characterized by reduced TJP expression may be the reason for colon epithelial injury following HIBD.

To date, previous studies regarding endogenous melatonin and melatonin receptors have indicated that there is a significant decrease in both the release and function of melatonin following HIBD, which suggests melatonin may function in the pathogenesis of HIBD. Moreover, exogenous melatonin supplementation can improve the stress state, exhibit beneficial effects in the maintenance of relative homeostasis, and improve the immunological functions of HIE neonates. Thus, it is necessary to further investigate the role of melatonin in the pathophysiological mechanisms of HIBD, such as edema in the central nervous system and peripheral organs. Our study provides timely and substantial evidence for the theoretical basis of melatonin application in the clinic.

Previous studies indicate that endogenous melatonin release decreased during the hypoxic-ischemic injury in rats, which may be involved in the pathogenesis of early HIBD [10, 12]. In addition, supplement of exogenous melatonin after hypoxic-ischemic injury is beneficial to protect neonatal perinatal brain damage [14]. In a prospective study melatonin combining with hypothermia promoted neonatal HIE survival and health by brain magnetic resonance imaging (MRI) analysis [25]. In another preterm brain injury study, small dose (0.1 mg/kg) of melatonin demonstrated a protective activity on pregnant ewe hyopoxic/ischemia injure model [26]. On the other hand, other study of melatonin treatment in an neonatal hypoxic-ischemic damage model did not show significant improvement in (1 h) early phase [27]. These results hint that different hypoxic/ischemia model and the method of melatonin employment may affect the result, as well as more longer observation is necessary. Our results did demonstrate a significant anti-edema damage activity in longer time course observation. In addition, our result provided a possible mechanism as well that melatonin indirectly regulates the edema related factors, AQP-4, ZO-1, and occludin, to partially protect hypoxic-ischemic injures.

Several mechanisms of melatonin may be involved in its effects. For example, based on its lipophilic and partly hydrophilic dissolubility, melatonin has a high diffusing capacity, which makes it easy to enter the central nervous system and act directly on cell membranes and nuclei [28]. It was believed that metalonin acts for neuroprotection and ischemia harm protection by its anti-oxidative and anti-apoptosis properties [29–33]. Thus, regarding the edema protection property, melatonin could change the transcriptional activity of edema related molecules, such as AQP-4 and TJPs, which is important for its improvement in brain edema. Furthermore, the melatonin receptor belongs to a G-protein-coupled receptor family, and exogenous melatonin can combine with its receptors soon after HIBD, which further activates the downstream MAPK signal pathway, especially the p38 MAPK and JNK/SAPK signal pathways [34, 35], both of which are important in the regulation of HIBD pathogenesis, such as edema related proteins. Moreover, exogenous melatonin could increase endogenous melatonin release and melatonin receptor regulation via the positive feedback path, as well as enlarge their effects via a cascade reaction. In addition, vascular endothelial growth factor (VEGF) has been reported to lead to the inactivation of TJPs, such as ZO-1, by decreasing their expression and increasing phosphorylation; previous studies have indicated that melatonin can improve VEGF release [36, 37], which indirectly regulates edema related protein expression. Additional research regarding the pathways involved in the regulation of edema related proteins for melatonin is necessary.

## Conclusions

In conclusion, our data indicate that melatonin treatment has protective effects on the brain and peripheral organs after HIBD. The edema related proteins, AQP-4, ZO-1, and occludin, may participate in the mechanism of edema protection by melatonin.

## Abbreviations

AQPs: Aquaporins
BBB: Blood-brain barrier
BWC: Brain water content
ER: Endoplasmic reticulum
HIBD: Hypoxic-ischemic brain damage
HIE: Hypoxic-ischemic encephalopathy
MT: Melatonin
qPCR: Real-time quantitative polymerase chain reaction
SD: Sprague Dawley
SPF: Specific pathogen free
TJ: Tight junctions
TJP: Tight junction proteins
VEGF: Vascular endothelial growth factor
ZO-1: Zonula occludens-1

## References

[1] Sarnat HB, Sarnat MS (1976). Neonatal encephalopathy following fetal distress. A clinical and electroencephalographic study. Arch Neurol.

[2] Volpe JJ (2000). Neurology of the Newborn. 4. Hypoxic-ischemic encephalopathy: Clinical aspects.

[3] Allen KA, Brandon DH (2011). Hypoxic ischemic encephalopathy: pathophysiology and experimental treatments. Newborn Infant Nurs Rev.

[4] Buonocore G, Perrone S, Turrisi G, Kramer BW, Balduini W (2012). New pharmacological approaches in infants with hypoxic-ischemic encephalopathy. Curr Pharm Des.

[5] Lupton BA, Hill A, Roland EH, Whitfield MF, Flodmark O (1988). Brain swelling in the asphyxiated term newborn: pathogenesis and outcome. Pediatrics.

[6] Wang X, Zhang J, Yang Y, Dong W, Wang F, Wang L (2013). Progesterone attenuates cerebral edema in neonatal rats with hypoxic-ischemic brain damage by inhibiting the expression of matrix metalloproteinase-9 and aquaporin-4. Exp Ther Med.

[7] Barnett CP, Perlman M, Ekert PG (1997). Clinicopathological correlations in postasphyxial organ damage: a donor organ perspective. Pediatrics.

[8] Scallan J, Huxley VH, Korthuis RJ. Capillary fluid exchange: regulation, functions, and pathology[J]. Colloquium. 2010(1). 21452435

[9] Liu K, Agre P. Aquaporin Water Channels. In: Lane WJLD, eidotr. Encyclopedia of Biological Chemistry. Waltham: Academic Press; 2013. p. 120-6.

[10] Anderson JM, Van Itallie CM (2009). Physiology and function of the tight junction. Cold Spring Harb Perspect Biol.

[11] Günzel D, Yu AS (2013). Claudins and the modulation of tight junction permeability. Physiol Rev.

[12] Alonso-Alconada D, Alvarez A, Arteaga O, Martinez-Ibarguen A, Hilario E (2013). Neuroprotective effect of melatonin: a novel therapy against perinatal hypoxia-ischemia. Int J Mol Sci.

[13] Brown GM (1994). Light, melatonin and the sleep-wake cycle. J Psychiatry Neurosci.

[14] Carloni S, Perrone S, Buonocore G, Longini M, Proietti F, Balduini W (2008). Melatonin protects from the long-term consequences of a neonatal hypoxic-ischemic brain injury in rats. J Pineal Res.

[15] Uz T, Giusti P, Franceschini D, Kharlamov A, Manev H (1996). Protective effect of melatonin against hippocampal DNA damage induced by intraperitoneal administration of kainate to rats. Neuroscience.

[16] Chen W, Jadhav V, Tang J, Zhang JH (2008). HIF-1alpha inhibition ameliorates neonatal brain injury in a rat pup hypoxic-ischemic model. Neurobiol Dis.

[17] Wang JW, Wang HD, Cong ZX, Zhang XS, Zhou XM, Zhang DD (2013). Activation of metabotropic glutamate receptor 5 reduces the secondary brain injury after traumatic brain injury in rats. Biochem Biophys Res Commun.

[18] Wang JW, Wang HD, Zhong WZ, Li N, Cong ZX (2012). Expression and cell distribution of metabotropic glutamate receptor 5 in the rat cortex following traumatic brain injury. Brain Res.

[19] Wang H, Wang X, Guo Q (2012). The correlation between DTI parameters and levels of AQP-4 in the early phases of cerebral edema after hypoxic-ischemic/reperfusion injury in piglets. Pediatr Radiol.

[20] Da T, Verkman AS (2004). Aquaporin-4 gene disruption in mice protects against impaired retinal function and cell death after ischemia. Invest Ophthalmol Vis Sci.

[21] Basile DP, Anderson MD, Sutton TA (2012). Pathophysiology of acute kidney injury. Compr Physiol.

[22] Wang W, Li C, Summer SN, Falk S, Wang W, Ljubanovic D (2008). Role of AQP1 in endotoxemia-induced acute kidney injury. Am J Physiol Renal Physiol.

[23] Yde J, Keely S, Wu Q (2016). Characterization of AQPs in Mouse, Rat, and Human Colon and Their Selective Regulation by Bile Acids. Front Nutr.

[24] Gonzalez-Mariscal L, Namorado MC, Martin D, Luna J, Alarcon L, Islas S (2000). Tight junction proteins ZO-1, ZO-2, and occludin along isolated renal tubules. Kidney Int.

[25] Aly H, Elmahdy H, El-Dib M, Rowisha M, Awny M, El-Gohary T (2015). Melatonin use for neuroprotection in perinatal asphyxia: a randomized controlled pilot study. J Perinatol.

[26] Drury PP, Davidson JO, Bennet L, Booth LC, Tan S, Fraser M (2014). Partial neural protection with prophylactic low-dose melatonin after asphyxia in preterm fetal sheep. J Cereb Blood Flow Metab.

[27] Berger HR, Morken TS, Vettukattil R, Brubakk AM, Sonnewald U, Wideroe M (2015). No improvement of neuronal metabolism in the reperfusion phase with melatonin treatment after hypoxic-ischemic brain injury in the neonatal rat. J Neurochem.

[28] Hardeland R (2015). Melatonin in plants and other phototrophs: advances and gaps concerning the diversity of functions. J Exp Bot.

[29] Gilad E, Cuzzocrea S, Zingarelli B, Salzman AL, Szabo C (1997). Melatonin is a scavenger of peroxynitrite. Life Sci.

[30] Hardeland R, Tan DX, Reiter RJ (2009). Kynuramines, metabolites of melatonin and other indoles: the resurrection of an almost forgotten class of biogenic amines. J Pineal Res.

[31] Wakatsuki A, Okatani Y, Izumiya C, Ikenoue N (1999). Melatonin protects against ischemia and reperfusion-induced oxidative lipid and DNA damage in fetal rat brain. J Pineal Res.

[32] Tan DX, Manchester LC, Terron MP, Flores LJ, Reiter RJ (2007). One molecule, many derivatives: a never-ending interaction of melatonin with reactive oxygen and nitrogen species?. J Pineal Res.

[33] Hassell KJ, Ezzati M, Alonso-Alconada D, Hausenloy DJ, Robertson NJ (2015). New horizons for newborn brain protection: enhancing endogenous neuroprotection. Arch Dis Child Fetal Neonatal Ed.

[34] Xu L, Pathak PS, Fukumura D (2004). Hypoxia-induced activation of p38 mitogen-activated protein kinase and phosphatidylinositol 3’-kinase signaling pathways contributes to expression of interleukin 8 in human ovarian carcinoma cells. Clin Cancer Res.

[35] Ferreira GM, Martinez M, Camargo IC, Domeniconi RF, Martinez FE, Chuffa LG (2014). Melatonin Attenuates Her-2, p38 MAPK, p-AKT, and mTOR Levels in Ovarian Carcinoma of Ethanol-Preferring Rats. J Cancer.

[36] Fischer S, Wobben M, Marti HH, Renz D, Schaper W (2002). Hypoxia-induced hyperpermeability in brain microvessel endothelial cells involves VEGF-mediated changes in the expression of zonula occludens-1. Microvasc Res.

[37] Kaur C, Sivakumar V, Ling EA (2010). Melatonin protects periventricular white matter from damage due to hypoxia. J Pineal Res.

